# Analysis of Clinical and Laboratory Profiles of Patients Hospitalized with Hemorrhagic Fever with Renal Syndrome in Southwestern South Korea

**DOI:** 10.4269/ajtmh.24-0019

**Published:** 2024-10-29

**Authors:** Ma Eum Park, Da Young Kim, Jun-Won Seo, Na Ra Yun, You Mi Lee, Choon Mee Kim, Dong-Min Kim

**Affiliations:** ^1^ School of Medicine, Chosun University, Dong-gu, Gwangju, South Korea;; ^2^ Department of Internal Medicine, College of Medicine, Chosun University, Gwangju, South Korea;; ^3^ Premedical Science, College of Medicine, Chosun University, Gwangju, South Korea

## Abstract

Hemorrhagic fever with renal syndrome (HFRS) is caused by hantaviruses. Data of 34 patients with HFRS hospitalized at Chosun University Hospital, South Korea, between 2010 and 2021 were retrospectively analyzed. Nested reverse transcription polymerase chain reaction (RT-nPCR) targeting the L segment of hantavirus and sequencing were used for diagnosis. Most cases occurred in men and during the months of October through December. Common symptoms were fever, chills, gastrointestinal symptoms, and myalgia. The common laboratory abnormalities were thrombocytopenia, proteinuria, and elevated levels of serum creatinine, aspartate transaminase, alanine transaminase, and lactate dehydrogenase. Approximately 91.2% of patients had the Hantaan virus with a new genotype cluster, whereas 8.8% had the Seoul virus. Seropositivity based on IgM titer >1:32 on admission was noted in 20.6%, and a 4-fold increase in IgG titer of 1:512 was observed in 11.8%. This study demonstrated that RT-nPCR targeting the L segment of hantaviruses is a more reliable diagnostic method compared to serological testing.

## INTRODUCTION

Hemorrhagic fever with renal syndrome (HFRS) is a rodent-borne viral disease characterized by the development of an acute febrile illness that may lead to hemorrhagic manifestations and renal failure.^1^ Other symptoms include myalgia, back pain, abdominal pain, nausea, vomiting, and diarrhea.^2^^,^^3^ There are five classical phases of HFRS: febrile, hypotensive, oliguric, diuretic, and convalescent.^1^^,^^4^ The causative agents of HFRS are hantaviruses, which are enveloped single-stranded RNA viruses belonging to the Orthohantavirus genus family *Hantaviridae*, and order Bunyavirales.^5^ Historically, the Hantaan virus (HTNV) accounted for approximately 70% of HFRS cases in South Korea, followed by the Seoul virus (SEOV) accounting for 20%.^6^ However, since the 21st century, new hantaviruses such as the Soochong virus (SOOV) and Muju virus have been detected. These viruses are considered the possible causes of HFRS cases that cannot be attributed to infections by HTNV or SEOV.^6^^–^^8^ In South Korea, HFRS shows a particular seasonal incidence pattern with the majority of cases reported in autumn (October to December) followed by early summer (May to July). This pattern may be associated with the breeding season of reservoir rodent hosts, climatic factors such as low humidity, and environmental exposure.^6^^,^^9^

Most studies on the clinical features of HFRS have been conducted on patients diagnosed via serologic tests.^10^ With the licensing and adoption of hantavirus vaccination among rural residents in endemic areas such as South Korea and China,^11^^,^^12^ diagnosing HFRS based solely on serologic tests and single antibody titers can lead to false positive results.^13^

Few studies have comprehensively described the clinical features of patients with HFRS confirmed by polymerase chain reaction (PCR), including genotype information, comparisons of immunofluorescence assay (IFA) test positivity rates with PCR, and the proportion of patients showing a 4-fold increase in antibody titers during their clinical course. This study aimed to describe the clinical and laboratory profiles of patients with HFRS and their clinical outcomes, and to compare the use of IFA and PCR for diagnosing hantaviruses at a teaching hospital in Gwangju, South Korea.

## MATERIALS AND METHODS

### Study design and population.

This observational study was conducted at Chosun University Hospital, a tertiary, 849-bed teaching hospital in Gwangju, the largest city in southwestern South Korea. Most patients treated in this hospital reside in Gwangju, Jeollanam-do, and Jeollabuk-do provinces of South Korea. This study included 34 patients with HFRS hospitalized between 2010 and 2021.

### Diagnosis of hantaviruses.

Hantavirus infections were diagnosed using nested reverse transcription PCR (RT-nPCR). Viral RNA was extracted from patients’ plasma (150 µL) or buffy coat (150 µL) using the Viral Genespin™ Viral DNA/RNA extraction kit (iNtRON, Seongnam, South Korea) according to the manufacturer’s protocol. RT-nPCR targeting the L segment of hantaviruses, including HTNV, SEOV, Dobrava–Belgrade virus, Puumala virus, SOOV, Sin Nombre virus, Andes virus, and Tula virus, was performed with recombinant complementary DNA produced by SuperScript VILO MasterMix (Invitrogen, Waltham, MA, USA). An RT-nPCR result was considered positive if the viral L segment (encoding viral RNA-dependent RNA polymerase) was detected.^14^

A phylogenetic tree was produced by neighbor-joining and maximum likelihood methods using the 360-bp amplification products of RT-nPCR targeting the Hantavirus L segments. The CLUSTAL X software (University College Dublin, Ireland) was used to construct the phylogenetic tree.

Serological assessment of immunoglobulin (Ig) M and IgG was performed using IFA slides containing the HTNV strain 76–118 obtained from the Korea Center for Disease Control and Prevention. The IFA titers were reported as the reciprocal of the highest dilution of serum that produced characteristic fluorescence in cells. Serum specimens were initially examined at 1:16 dilution, followed by serial 2-fold dilutions to determine the endpoint titer.

## STATISTICAL ANALYSES

Clinical data, including demographic features, clinical manifestations, complications, and laboratory test results were retrospectively collected from patient medical records. All statistical analyses were performed using the SPSS software (version 26.0; SPSS Inc., Chicago, IL, USA). Data are described as means ± SD or medians for continuous variables and as percentages for categorical variables. Risk factor analysis was conducted using univariate regression analysis to explore the relationship between laboratory results on admission and outcomes such as intensive care unit (ICU) admission or death.

### Ethics approval.

This study was approved by the Ethics in Human Research Committee of Chosun University Hospital (IRB no. 2013-10-001–018), Gwangju, South Korea. All patient data were anonymized prior to analysis.

## RESULTS

**Figure 1.**
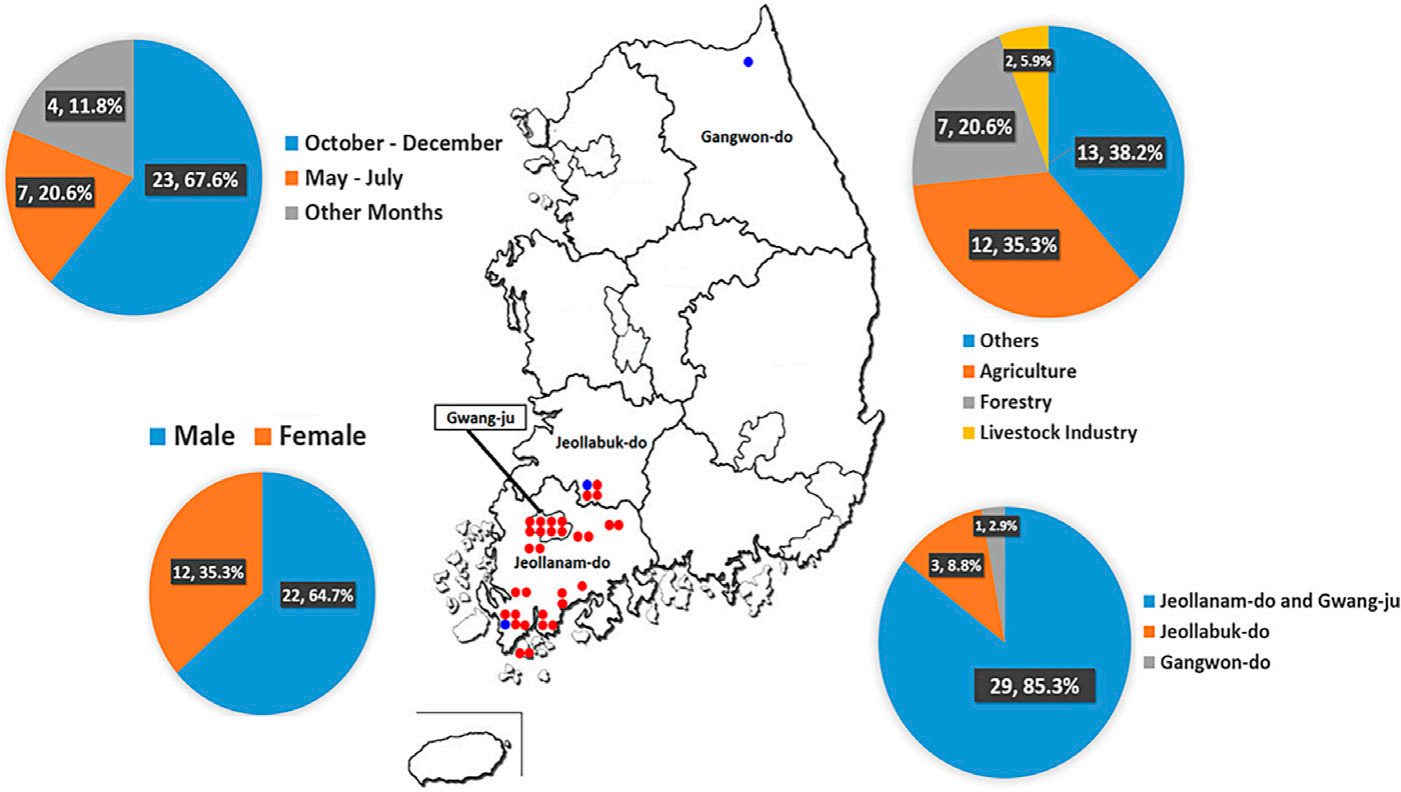
The demographic data of 34 patients with HFRS who were treated at a tertiary hospital in Gwangju. Four patients who were infected in other months had HFRS in January, March, April, and August, respectively. The presumed infection locations were marked in dots. Red dots indicate HTNV infection, and blue dots indicate SEOV infection.

A total of 34 patients with HFRS were hospitalized during the study period. The majority of the patients were from the southwestern part of South Korea, except for one case (Figure 1). Of the 34 total patients, there were 22 (64.7%) males and 12 (35.3%) females; 21 (61.8%) patients worked in agriculture, forestry, or livestock industries. The median age of the patients was 56 years (range, 18–87 years) (Table 1). Most cases occurred during the months of October through December (67.6%) and May through July (20.6%). Twelve patients (35.3%), at the time of infection, had underlying chronic disease; eight with hypertension, and the remaining four with other conditions such as diabetes, breast cancer, and chronic hepatitis B infection.

**Table 1:** Clinical manifestations of patients with HFRS on hospital admission

Clinical Manifestations	Number (%) (*N* = 34)
Male	22 (64.7)
Female	12 (35.3)
Median age (years)	56 (range 18–87)
Symptom onset to first laboratory testing (days)	5.8±3.5 (range 2–16)
Underlying chronic disease	12 (35.3)
General symptoms	
Fever	22 (64.7)
Chills	20 (58.8)
Myalgia	16 (47.1)
Neck stiffness	1 (2.9)
Headache	13 (38.2)
Rash	1 (2.9)
Hypertension during hospitalization	27 (79.4)
Hypotension during hospitalization	16 (47.1)
GI symptoms	20 (58.8)
Anorexia	12 (35.3)
Nausea	12 (35.3)
Vomiting	9 (26.5)
Diarrhea	13 (38.2)
Dyspepsia	5 (14.7)
Abdominal pain	13 (38.2)
Renal symptoms	
Microscopic hematuria	12 (35.3)
Macroscopic hematuria	0 (0)
Proteinuria (>+1) on hospital arrival	26 (76.5)
Proteinuria (>+1) during hospitalization	28 (82.3)
Proteinuria (>300 mg/d) on hospital arrival	22 (64.7)
Proteinuria (>300 mg/d) during hospitalization	22 (64.7)
Oliguria (<0.5 mL/kg/h) on hospital arrival	4 (11.8)
Oliguria (<0.5 mL/kg/h) during hospitalization	8 (23.5)
Polyuria (≧3 mL/kg/h) during hospitalization	11 (32.4)

### Clinical and laboratory features and outcomes.

The mean interval from symptom onset to hospital visit was 5.8 ± 3.5 days (range, 2–16 days). The common symptoms observed upon admission included fever (64.7%), chills (58.8%), myalgia (47.1%), and headache (38.2%). During hospitalization, proteinuria greater than +1 was noted in 82.3% of the cases, and proteinuria >300 mg persisted in 64.7% of the cases. On admission, 11.8% of the patients presented with oliguria, while during hospital stay, 23.5% developed oliguria and 32.4% had polyuria. Table 1 provides detailed information on the clinical manifestations of patients with HFRS upon hospital admission.

The laboratory investigation results obtained on admission and during hospitalization are presented in Table 2. Thrombocytopenia (94.1%), elevated liver marker (aspartate aminotransferase [AST] >40 IU/L, 91.2%; alanine aminotransferase [ALT] >40 IU/L, 58.5%) and lactate dehydrogenase levels (LDH) (100%), and decreased albumin levels (76.5%) were the common laboratory abnormalities observed. Severe thrombocytopenia with a platelet count <50,000/µL was documented in 16 (47.1%) patients. Leukocytosis was reported in 19 (55.9%) patients on admission and 23 (67.6%) patients during hospitalization. Increased creatinine levels >1.2 mg/dL were observed in 27 (79.4%) patients, whereas 7 (20.6%) patients did not show any abnormalities in creatinine levels during hospitalization. The median C-reactive protein (CRP) level was 5.7 mg/dL on admission, and 97.1% patients had increased CRP levels >1.0 mg/dL.

**Table 2:** Laboratory parameters of patients HFRS patients on admission and during hospitalization

Parameters	Number (%)		Normal Range
	On Admission	During Hospitalization	
WBC (/μL)>10,000	19 (55.9)	23 (67.6)	4,000–10,000
WBC (/μL)<4,000	2 (5.9)	5 (14.7)	4,000–10,000
Platelet (×1,000/μL)<50	16 (47.1)	19 (55.9)	150–400
Platelet (×1,000/μL)<150	32 (94.1)	33 (97.1)	150–400
Hemoglobin (g/dL)<11	4 (11.8)	17 (50.0)	11.0–15.0
Hemoglobin (g/dL)>15	12 (35.3)	19 (55.9)	11.0–15.0
Hematocrit (%)>45	8 (23.5)	14 (41.2)	35–45
Albumin (g/dL)<3.5	26 (76.5)	31 (91.2)	3.5–5.3
AST (IU/L)>40	31 (91.2)	34 (100)	10–40
AST (IU/L)>200	10 (29.4)	12 (35.3)	10–40
ALT (IU/L)>40	20 (58.8)	27 (79.4)	10-40
ALT (IU/L)>200	5 (14.7)	6 (17.6)	10–40
PT (sec)>14	4 (12.1)^a^	5 (14.7)	12–14
aPTT (sec)>45	10 (30.3)^b^	13 (38.2)	33–45
LDH (U/L)>300	16 (100)^c^	24 (70.6)	140–280
Urine specific gravity>1.030	10 (29.4)	2 (6.9)^d^	1.000–1.030
BUN (mg/dL)>23	23 (67.6)	26 (76.5)	8.0–23.0
Creatinine (mg/dL)>1.2	23 (67.6)	27 (79.4)	0.5–1.2
eGFR (mL/min/1.73m^2^)	22 (64.7)	27 (79.4)	>60
CRP (mg/dL)>1.0	9 (26.5)	33 (97.1)	<1.0

*Abbreviation*: ALT = alanine aminotransferase; aPTT = activated partial thromboplastin time; AST = aspartate aminotransferase; BUN = blood urea nitrogen; CRP = C-reactive protein; eGFR = estimated glomerular filtration rate; LDH = lactate dehydrogenase; PT = prothrombin time.

^a^ Data was only available for 33 among 34 patients.

^b^ Data was only available for 33 among 34 patients.

^c^ Data was only available for 16 among 34 patients.

^d^ Data was only available for 29 among 34 patients.

The average duration of hospitalization for patients was 13 days (range, 3–35 days) (Table 3). Nine patients (26.5%) required ICU admission, and 10 patients (29.4%) underwent hemodialysis. Eight patients presented thrombocytopenia and renal dysfunction, six patients presented with hypotension, and an APACHE II score greater than 10 in 5 patients, and the need for continuous renal replacement therapy (CRRT) was seen in three patients. Some patients experienced multiple complications.

**Table 3:** Clinical outcomes of patients with HFRS

Clinical Outcomes	Number (%) (*N* = 34)
Duration of hospitalization (days)	13 (mean, range 3–35)
Intensive care unit admission	9 (26.5)
Hemodialysis	10 (29.4)
Duration of hemodialysis (days)	5 (mean, range 2–11)
Death	1 (2.9)

Of the 34 patients, one patient died of multiorgan failure after undergoing an appendectomy for acute appendicitis related to HFRS.^15^ Ten (29.4%) patients required hemodialysis for a mean duration of 5 days (range, 2–11 days).

### Confirmatory laboratory tests and genotypes.

IFA results on admission revealed that the number of patients with an IgM titer of ≥1:16 was 11 (32.4%) (Table 4). The number of patients with an IgM titer of ≥1:32 was seven (20.6%). IgG titers of 1:32 were observed in 24 (70.6%) patients. IgG titers of 1:512 were observed in 11 (32.4%) patients. Among the 28 (82.4%) patients who underwent follow-up IFA tests throughout the disease course, 4-fold titer increases in IgM and IgG were observed in 7 (20.6%) patients and 21 (61.8%) patients, respectively. Among the seven (20.6%) patients with 4-fold titer increases in IgM, four (11.8%) showed an increase in titer of ≥1:512.

**Table 4:** Comparison of RT-nPCR for L-segment and serological testing with IFA for diagnosis of hantaviruses in patients hospitalized with HFRS

Patient	Sex	Age	RT-nPCR	On Admission IFA		Follow-Up		4-Fold Increase
				Day^a^	IFA^b^ IgG/IgM	Day^c^	IFA^d^ IgG/IgM	
1	M	60	SEOV	16	1:64/<1:16	18	1:1,024/<1:16	+/−
2	M	46	SEOV	6	1:512/<1:16	13	1:1,024/<1:16	−/−
3	F	34	SEOV	4	1:128/<1:16	10	>1:1,024/<1:16	+/−
4	F	57	HTNV	10	1:32/<1:16			
5	M	68	HTNV	13	<1:16/<1:16			
6	M	54	HTNV	2	1:64:<1:16	10	>1:2,048/<1:16	+/−
7	M	43	HTNV	8	1:64/<1:16			
8	M	54	HTNV	6	<1:16/<1:16	12	1:512/<1:16	+/−
9	F	66	HTNV	9	1:128/<1:16			
10	F	69	HTNV	4	<1:16/<1:16	8	1:128/<1:16	+/−
11	M	87	HTNV	5	<1:16/<1:16	10	<1:16/<1:16	−/−
12	F	77	HTNV	3	<1:16/<1:16	8	<1:16/<1:16	−/−
13	F	66	HTNV	4	<1:16/<1:16			
14	M	67	HTNV	15	1:32/<1:16	18	1:512/<1:16	+/−
15	M	83	HTNV	6	1:256/<1:16	7	1:1,024/1:256	+/+
16	F	79	HTNV	3	1:64/1:16	7	>1:512/1:256	+/+
17	M	66	HTNV	4	<1:16/<1:16	7	1:128/<1:16	+/−
18	M	51	HTNV	5	1:2,048/−	11	>1:2,048/>1:256	−/−
19	F	39	HTNV	6	1:128/<1:16	11	1:4,096/1:512	+/+
20	M	24	HTNV	4	1:512/1:32	10	1:2,048/1:256	+/+
21	M	41	HTNV	7	1:32/<1:16	43	1:2,048/1:32	+/−
22	M	56	HTNV	3	1:1,024/<1:16	7	1:2,048/<1:16	−/−
23	F	77	HTNV	3	1:512/1:64	19	1:2,048/1:32	+/−
24	M	28	HTNV	6	<1:16/<1:16			
25	M	67	HTNV	3	1:256/1:128	10	1:1,024/<1:16	+/−
26	F	67	HTNV	3	1:1,024/<1:16	9	1:4,096/<1:16	+/−
27	F	37	HTNV	3	1:512/1:16	6	1:8,192/<1:16	+/−
28	M	52	HTNV	8	1:512/<1:16	26	1:2,048/1:512	+/+
29	M	54	HTNV	4	1:512/1:512	13	1:4,096/<1:16	+/−
30	F	46	HTNV	3	<1:16/<1:16	12	1:2,048/1:1,024	+/+
31	M	56	HTNV	5	1:64/1:64	23	1:4,096/1:512	+/+
32	M	58	HTNV	4	1:2,048/1:64	15	>1:4,096/<1:16	−/−
33	M	18	HTNV	9	>1:4,096/1:1,024	16	>1:4,096/1:512	−/−
34	M	46	HTNV	7	1:16/<1:16	14	1:1,024/<1:16	+/−

*Abbreviation*: IFA = immunofluorescence assay; HTNV = Hantaan virus; RT-nPCR = nested reverse transcription polymerase chain reaction; SEOV = Seoul virus.

^a^ Interval (day) between symptom onset and first confirmatory testing.

^b^ Value measured on first confirmatory testing.

^c^ Interval (day) between symptom onset and peak value testing.

^d^ Peak value during hospitalization.

**Figure 2.**
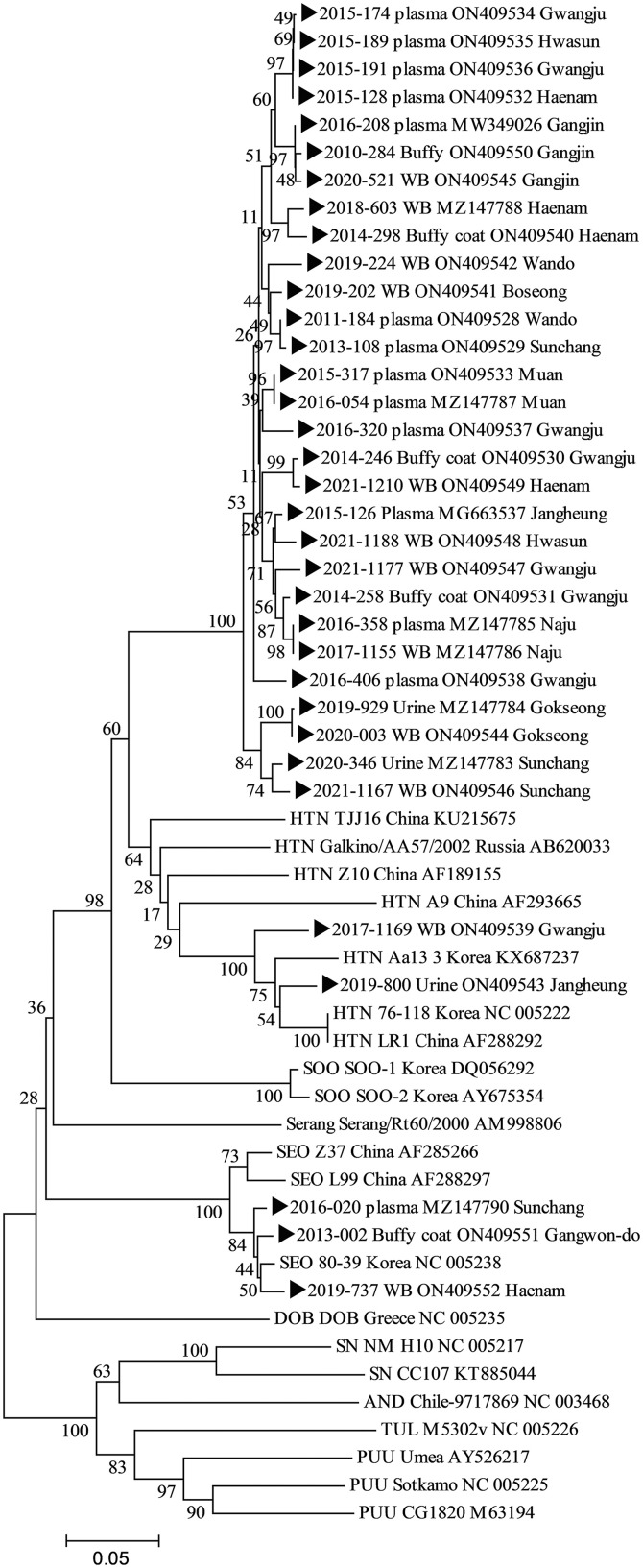
Phylogenetic trees for hantaviruses based on the partial L-segment genome sequences (360nt). CLUSTAL X software program was used to construct the phylogenetic trees using the neighbor-joining (NJ) with 1,000 bootstrap replicates. AND = Andes virus; DOB = Dobrava–Belgrade virus; HTN = Hantaan virus; PUU = Puumala virus; SEO = Seoul virus; SN = Sin Nombre virus; SOO = Soochong virus; TUL = Tula virus.

RT-nPCR identified HTNV in 31 patients (91.2%) and SEOV in 3 patients (8.8%) (Table 4). All three specimens from the SEOV cases were clustered with the SEOV 80-39 Korea NC005238. We identified a new genotype cluster that included 29 HTNV specimens from South Korea. Two specimens were clustered with HTN Aal3 3 KoreaKX687237 (Figure 2).

## DISCUSSION

This study describes the clinical and laboratory features of patients hospitalized for HFRS with confirmed diagnoses of HTNV or SEOV using nPCR. A review of 35 cases of HFRS detected in South Korea between 2002 and 2012 reported a history of fever in 97.1% and fever at the time of hospital admission in 45.7% of the patients.^6^ Similar findings were observed in our study, where 64.7% of the patients had fever during hospitalization. Additionally, gastrointestinal symptoms such as nausea, vomiting, diarrhea, and abdominal pain were frequently reported.^6^ Renal involvement manifested as hematuria, proteinuria, acute kidney injury, and polyuria. In our study, 79.4% of the patients exhibited elevated serum creatinine levels, 23.5% developed oliguria during hospitalization, and five patients required renal replacement therapy. Nearly half of the patients experienced hypotension upon admission, with some requiring ICU admission and vasopressor infusion. These clinical features underscore the potential for life-threatening complications in patients with HFRS. Therefore, prompt and appropriate management of symptoms is key in reducing mortality in these patients.

The incidence of HFRS is remarkably higher in the months of October, November, and December when more outdoor and farming activities occur.^4^^,^^16^^,^^17^ However, a lack of seasonal variation in the occurrence of HFRS in Yeonchoen region, located in the north-central part of South Korea has been reported.^9^ In our study, the majority of the patients had been hospitalized between October and December. This seasonal variation may highlight the importance of epidemiological factors, environmental exposure, and virus genotypes in determining disease severity and patient outcomes.

The case fatality rate of HFRS has decreased from 5–7% in the 1950s to 1% in data representative up to 2016.^6^ Bimodal distribution with higher mortality rates has been reported among the age groups 10–19 and 40–49 years.^4^ The common clinical factors among non-survivors of HFRS include multiorgan dysfunction, shock, cerebral edema, cerebral hemorrhage, arrhythmia, gastrointestinal bleeding, and acute kidney injury.^18^ In our study, only one patient that had an additional surgical condition died.^15^ The common laboratory parameters in non-survivors include leukocytosis, thrombocytopenia, and increased levels of LDH, ALT, and AST.^18^

Serological tests are the primary diagnostic methods for identifying acute or past infections caused by hantaviruses.^19^^,^^20^ A study using Sin Nombre virus-specific enzyme-linked immunosorbent assay tests has demonstrated an early peak of IgM antibodies in all patients and a higher titer of IgG response among survivors with hantavirus pulmonary syndrome in the US.^19^ Point-of-care rapid tests utilizing recombinant neucleocapsid protein antigens from Puumala, Dobrava, and Hantaan (strain 76–118) viruses showed sensitivity for detecting IgM antibodies when using single antigens.^20^ However, combining these antigens was not recommended because of a significant risk of cross-reactivity.

By the time symptoms are evident, patients uniformly have IgM antibodies, and most also produce IgG antibodies. Korea Disease Control and Prevention Agency does not suggest a cut-off value owing to a lack of epidemiological data, and the threshold for distinguishing between vaccine-induced antibody titers and acute infection remains unknown. Therefore, in our cohort, we included patients with infections that were confirmed by nested PCR. In this study, only seven (20.6%) patients were IgM-positive for HFRS on admission at a cutoff value of ≥1:32 using the IFA test. On admission, an IgG titer of >1:512 was observed in 11 (32.4%) patients. A 4-fold titer increases were detected only in 61.8% of the patients. This demonstrates the limitations of IFA in diagnosing hantaviruses considering variable profiles on serological testing. In addition, commercialized enzyme-linked immunosorbent assays are currently not available in South Korea.

RT-qPCR has high sensitivity and specificity in detecting various genotypes of the hantavirus group.^1^ The use of RT-nPCR targeting the large (L) segment of hantaviruses was particularly useful in establishing early diagnosis of the illness, and the virus was detected in urine and serum samples up to 1 month after initial presentation.^14^ Compared with serological testing, RT-nPCR is considered a more reliable method of diagnosis, especially because of the variable serological profiles as demonstrated in this study.

Although HTNV strain 76–118 is a common genotype frequently encountered in patients with HFRS worldwide,^21^ this study identified a new genotype cluster in southern South Korea. However, this finding was based on the partial L-segment of the genome involving only 360 bp. Thus, this genotype should be further investigated, including next-generation sequencing.

This study had some limitations. Detailed information, such as vaccination history or specific outdoor exposure near the infection site, were unavailable because of the retrospective study design based on medical records. In addition, as this study was performed in a single hospital, the results may be insufficient to represent the HFRS trends in southwestern Korea. Further studies are necessary to analyze the distribution of viral genotype and their clinical implications. Collaborative studies conducted in multiple hospitals are required to improve our understanding of HFRS in South Korea.

## CONCLUSION

In conclusion, using RT-nPCR against the L segment of hantaviruses is a more reliable diagnostic method compared with serological testing. In this study, the majority of the patients with HFRS were infected with the HTNV, and only three were infected with the SEOV. A new genotype cluster of HTNV was observed among patients in this study. Future collaborative studies should describe the clinical, epidemiological, and virological genotype of hantaviruses to enhance our understanding of HFRS in South Korea.
